# The relationship between ethical climate and nursing service behavior in public and private hospitals: a cross-sectional study in China

**DOI:** 10.1186/s12912-021-00655-7

**Published:** 2021-08-05

**Authors:** Na Zhang, Jingjing Li, Xing Bu, Zhen-Xing Gong

**Affiliations:** 1 School of Economics and Management, Beijing Information Science & Technology University, Beijing, China; 2 College of Economics and Management, Beijing University of Technology, Beijing, China; 3 School of Economics and Management, University of Science and Technology Beijing, Beijing, China; 4 School of Business, Liaocheng University, Liaocheng, China

**Keywords:** In-role service behavior, Extra-role service behavior, Ethical climate, Hospital ownership

## Abstract

**Background:**

Workplace climate is a great significant element that has an impact on nurses’ behavior and practice; moreover, nurses’ service behavior contributes to the patients’ satisfaction and subsequently to the long-term success of hospitals. Few studies explore how different types of organizational ethical climate encourage nurses to engage in both in-role and extra-role service behaviors, especially in comparing the influencing process between public and private hospitals. This study aimed to compare the relationship between the five types of ethical climate and nurses’ in-role and extra-role service behaviors in public and private hospitals.

**Methods:**

This study conducted a cross-sectional survey on 559 nurses from China in May 2019. The questionnaire was distributed to nurses by sending a web link via the mobile phone application WeChat through snowball sampling methods. All participants were investigated using the Ethical Climate Scale and Service Behavior Questionnaire. SPSS 22.0 was used for correlation analysis, t-test, and analysis of variance test, and Mplus 7.4 was used for group comparison (*p* < .05).

**Results:**

The law and code climate has a much greater influence on nurses’ in-role service behavior in private hospitals than on that in public hospitals (*β* = − 0.277; CI _95 %_ = [-0.452, − 0.075]; *p* < .01), and the instrumental climate has a stronger influence on nurses’ extra-role service behavior private hospitals than on that in public hospitals (*β* = − 0.352; CI _95 %_ = [-0.651, − 0.056]; *p* < .05). Meanwhile, the rules climate has a greater effect on nurses’ extra-role service behavior in public hospitals than it does in private hospitals (*β* = 0.397; CI 95 % = [0.120, 0.651]; *p* < .01).

**Conclusions:**

As the relationship between the five types of ethical climate and nurses’ in-role and extra-role service behaviors in public and private hospitals were different, the strategies used to foster and enhance the types of ethical climate are various from public to private hospitals. The caring and instrumental climate are the key to promote extra-role service behavior for nurses in private hospitals. And the independent climate has a great effect on extra-role service behaviors for nurses in public hospitals.

**Supplementary Information:**

The online version contains supplementary material available at 10.1186/s12912-021-00655-7.

## Background

As there is considerable emphasis on the provision of patient-centered service in all aspects of health care [1], growing research attention is being devoted to factors contributing to providing high- quality service [2]. Empirical evidence shows that, to the extent that nurses are able to deliver high-quality care and service, patients are more likely to generate favorable evaluations of service encounters and experience higher satisfaction [3, 4], which, in turn, affects organizational effectiveness and performance [5]. Therefore, nurses’ service behaviors have critical implications for hospitals.

The service behavior of nurses is composed of moral philosophy with core values, including the consideration of the patient’s wants, needs, and preferences [6]. Hence, nursing has been considered to be an “ethical laden practice” [7]. Nevertheless, previous researchers have neglected the ethical aspects of nursing service practices [8]. Therefore, researchers should explore the promotion factors of nurse’ service behavior from the ethical aspects in hospitals, such as ethical climate.

Additionally, previous studies on customer service behavior focused on two types of employee behavior, including in-role behavior and extra-role behavior [9–11]. The in-role service behavior refers to the required or expected customer service behavior that stems from hidden rules in the workplace or clear duties and responsibilities expressed in company regulations, such as job descriptions and key performance indicators [12]. While the extra-role service behavior is the employee’s informal behavior that goes beyond the formal job requirements in serving customers [11, 13].

The existing evidence indicates that many service-oriented behaviors of frontline employees can be conceptualized as forms of organizational citizenship behavior (OCB) [14, 15]. OCB represents those organization-directed behaviors that go “above and beyond” normal task performance [16]. However, previous researches of OCB predominantly focus on behaviors that are directed at other coworkers or at the organization in general [15], such as helping colleagues and speaking positively about the organization. Extra-role service behavior is a kind of customer-focused behavior in service organizations, and its conceptualization has been expanded to include behaviors directed at customers. Therefore, OCB and extra-role service behavior are distinguished based on the recipient or beneficiary of the intended non-requisite actions.

Both the in-role and extra-role service behaviors are discretionary. They all have a strong flavor of service orientation that involves satisfying customers’ work-related problems. Thus, it is critical that the researcher should consider both of the two types of service behavior when studying the antecedents, outcomes, and mechanism of service behavior, because they may have different impact results and paths [12, 17].

### Ethical climate and service behavior

Defined by Victor and Cullen [18, 19], the ethical climate is “the shared perceptions of what ethically correct behavior is and how ethical issues should be handled,” and they believed that ethical climate can influence employee behavior [19]. Based on the theory proposed by Rodney et al. [20], the ethical climate in the health care field means the “implicit and explicit values that drive healthcare delivery and shape the workplaces in which care is delivered” [21]. According to Numminen et al. [22], the definition of the ethical climate of nursing is the perceptions of nurses about dealing with ethical issues in the workplace. Nursing practice is carried out in a social framework in which environmental factors and interpersonal relationships interact with each other [22]. Therefore, the climate of the work environment is crucial for nurses’ behavior and nursing practice [23]. As a kind of organizational climate, the ethical climate significantly affects the professional and ethical practice of nurses, and as a result, it should be attached importance to in evaluating nurses’ service behaviors.

According to the level of moral development of the workgroup (egoism, benevolence, and principled), Victor and Cullen [18, 19] developed a typology of ethical climates that the locus of analysis was used for final decisions (individual, local and cosmopolitan). Building on this typology, the five most common climate types in the research process include “caring” (employees’ behaviors and decisions should focus on the well-being of others),“law and code” (whether employees’ behavior and decisions are contrary to legal norms or principles),“rules” (employees should consider whether their behaviors and decisions disobey company rules and regulations),“instrumental” (organizations provide a climate concerning employees’ self-interest), and “independent” (organizations provide a climate that employee can act or make decisions depending on their personal ethical beliefs) [24, 25].

However, the literature is largely silent on how organizational ethical climate affects nurses to imply service behaviors. According to the current literature, the ethical climate not only affects which issues organizational members consider to be ethics-related, but also plays a decisive role in the generation of moral standards that organization members can understand, weigh and solve these problems [24]. On one hand, the ethical climate works through formal culture. Formal culture refers to the aspects such as leadership, structures, reward systems, policies, decision-making processes, and socialization mechanisms [22]. This, therefore, suggests that the ethical climate may play a role in facilitating employees’ in-role service behavior. On the other hand, the ethical climate also plays an important role in the informal atmosphere, such as in languages, role models, behavioral norms, rituals, and historical anecdotes [22, 26], which suggests that the ethical climate will lead to a higher level of extra-role service behavior. In the year of 1975, Schneider strongly believed that because of the diversity of climate types that exist within an organization, it is imperative that researchers focus on those dimensions of climate that are associated with specific variables, rather than focusing on the climate in general [27]. However, evidence specific to the effect of different ethical climate types on nurses’ in-role service behaviors and extra-role service behaviors remains limited.

### Hospital ownership and service behavior

In recent ten years, private hospitals in China have developed rapidly, but they are in fact designated as the supplement of public hospitals [28]. Because public hospitals account for the majority of hospitals in China, and they play vital roles in keeping citizen’s health and responding to the emergency public health crisis, such as the COVID-19 pandemic. Of course, the public hospitals in China have significant medical resource advantages, public hospitals are in a better position to recruit health care workers and provide services [28]. In China, most private hospitals are primarily profit-driven, and for this reason, it makes sense that private hospital services are largely determined by the market. Private hospitals adopt the marketing concept and seek to attract patients by concerning patients’ satisfaction and providing consumer-oriented services [29]. In addition, there are different employment contracts for nurses in public and private hospitals, such as permanent contract, fixed term contract, and agency employment contract [29]. Various types of contracts not only present different salary and compensation systems, but also different job requirements for nurses to stay in their organizations. Therefore, nurses in different ownership hospitals may act differently in nursing service behaviors.

Additionally, the difference in hospital ownership implies potentially different resources, operation modes, and climates. The issue as to whether hospital ownership has an impact on the quality of nursing service has long been a serious concern [30]. Some researchers argue that nurses in public hospitals perform better than those in private hospitals [31, 32]. However, others argue the reverse conclusions [33]. Since the nurses’ service behavior contains the moral philosophy with core values that considering patient’s wants, needs, and preferences [6], ethical climate may be the direct and effective explanations of service behavior and can clarify the mixed associations between hospital ownership and nursing service behavior. The hospital ownership may moderate the relationships between ethical climate and nurses’ service behavior, especially in China. Considering hospital ownership has both the theoretical and practical contributions in the study of ethical climate and service behavior, that help researchers find a key factor for service behavior research and inspire nursing managers to propose down-to-earth managerial solutions.

## Methods

### Aim

 This research aims to compare the links between various ethical climate types and nurses’ in-role and extra-role service behaviors in public and private hospitals.

### Design and sample

A pilot test was conducted for the initial questionnaire after being developed by three doctors in management and nursing. Based on the type of related questions, the length of the questions, the answer options, and the time to complete the questionnaire in the feedback, the questionnaire was modified and improved, resulting in the final questionnaire.

Participants were reached by the mobile phone application (APP) WeChat. WeChat is the most frequently used social networking tool by Chinese individuals [34]. Therefore, it is convenient and widely used to conduct surveys via WeChat. In May 2019, the questionnaire was sent to nurses through the third author’s WeChat account. Using the snowball sampling method, each respondent was asked to share the questionnaire with their nurse colleagues via WeChat. There were 620 nurses from 13 provinces in China who clicked the web link of the questionnaire. They fully completed the online survey and sent back their questionnaires at last. After deleting 61 invalid questionnaires with duplicate IP addresses in responses and short completion times (under 200 s), there were 559 valid questionnaires in total (response rate: 90.2 %).

### Measures

All measurements were made in Chinese. Since the relevant concept of ethical climate was originally produced in English, a back-translation procedure from English to Chinese was used [35], and there were no significant linguistic differences between these two sets of concepts. Additionally, all methods were performed in accordance with the relevant guidelines and regulations.

#### Ethical climate

Following Abou’s [36] research, the ethical climate was measured using the questionnaire introduced and authorized by Cullen et al.[37], which was completed to measure nurses’ views on the ethical atmosphere of their affiliations. As presented in Additional file 1, the scale comprised 24 items and is divided into five dimensions: caring (5 items), law and code (4 items), rules (4 items), instrumental (6 items), and independent (5 items). We measured the outcomes using a 5-point Likert scale (1 = strongly disagree to 5 = strongly agree). The higher the ethical climate score, the higher the nurse’s perception of the ethical climate. The internal consistency, measured using Cronbach’s α, was 0.865, 0.886, 0.853, 0.881, and 0.853 for caring, professional, rules, instrumental and independent, respectively. Cronbach’s α for the whole scale of ethical climate was 0.817.

#### Service behavior

 As presented in Additional file 1, an eight-item scale developed by Chen [38] was adopted to measure nurses’ service behaviors, and two dimensions were included in the scale, consisting of in-role service behaviors and extra-role service behaviors. In this study, the authorized nurses’ service behavior scale was used [39]. All the survey items were rated on a 5-point Likert scale (1 = strongly disagree to 5 = strongly agree). A higher score means the level of nurses’ service behavior can be higher. Cronbach’s α for in-role service behavior and extra-role service behavior was 0.923 and 0.835, respectively. Cronbach’s α for the whole scale of service behavior was 0.906.

### Data analysis

SPSS 22.0 statistical software packages were first utilized for data analysis. Firstly, the demographic characteristics of the sample were described with mean (M), standard deviation (SD), number (n), and percentage (%) as appropriate. Group differences of continuous variables were tested by t-test or one-way ANOVA. We then presented the means, standard deviations, and correlation values among the study variables. As hospital ownership is a binary variable, whether public or private hospitals, in the end, we used group comparison to compare the link between the ethical climate types and the service behaviors of nurses in public and private hospitals, respectively with Mplus 7.4.

## Results

### Descriptive statistics

Of all the nurses who participated in the survey, 94.7 % were female, were of young age (88.7 % under 40 years of age) and had college education (51.1 % with a bachelor’s degree or higher). In terms of hospital ownership, 70.3 % were from the public hospital, and 29.7 % were from the private hospital. The respondents’ demographic information and group differences on in-role and extra-role service behaviors are described in detail in Table 1. Participants with high level of in-role service behaviors were > 40 years old (*p* < .001), worked for 11–15 years (*p* < .001), had bachelor’s degree (*p* < .001), had senior nurse professional title (*p* < .001), temporary employed (*p* < .001) and were from public hospitals (*p* < .001). Nurses who had higher level of extra-role service behaviors were > 40 years old (*p* < .001), worked for 16–20 years (*p* < .001), had master’s degree or above (*p* < .001), had junior nurse professional title (*p* < .001), and temporary employed (*p* < .001).

**Table 1:** Demographic characteristics of the sample (N = 559) and comparisons of service behavior

Demographics	n	%	In-role service behaviors			Extra-role service behaviors		
			**M ± SD**	***F/t***	***p***	**M ± SD**	***F/t***	***p***
**Gender**				0.410	0.522		0.578	0.447
Female	523	94.7	4.16 ± 0.93			3.64 ± 1.03		
Male	36	5.3	4.06 ± 0.89			3.50 ± 1.16		
**Clinical tenure (years)**				21.828	0.000		7.150	0.000
≤ 5	290	51.9	3.83 ± 1.11			3.43 ± 1.12		
6–10	148	26.5	4.51 ± 0.53			3.72 ± 0.95		
11–15	46	8.2	4.54 ± 0.45			4.00 ± 0.68		
16–20	19	3.4	4.43 ± 0.31			4.16 ± 0.64		
> 20	56	10.0	4.53 ± 0.37			3.92 ± 0.92		
**Age**				478.859	0.000		91.140	0.000
≤ 20	58	7.1	1.84 ± 0.48			1.90 ± 0.45		
21–30	309	57.4	4.40 ± 0.50			3.79 ± 0.90		
31–40	129	25.4	4.46 ± 0.52			3.78 ± 0.93		
> 40	63	10.1	4.48 ± 0.41			4.08 ± 0.74		
**Education level**				65.968	0.000		15.140	0.000
Certificate (technical school)	102	18.2	3.17 ± 1.27			3.10 ± 1.22		
Junior college	171	30.6	4.26 ± 0.92			3.56 ± 1.06		
Bachelor’s degree	274	49.0	4.46 ± 0.42			3.84 ± 0.88		
Master’s degree or above	12	2.1	4.22 ± 0.39			4.19 ± 0.58		
**Professional title**				43.352	0.000		7.610	0.001
Primary nurse	289	51.7	3.83 ± 1.12			3.46 ± 1.12		
Junior nurse	178	31.8	4.50 ± 0.49			3.81 ± 0.96		
Senior nurse	92	16.5	4.54 ± 0.42			3.79 ± 0.82		
**Position**				1.671	0.189		0.122	0.885
General nurse	180	32.2	4.09 ± 1.04			3.63 ± 1.02		
Unit manager	296	53.0	4.22 ± 0.84			3.61 ± 1.02		
Supervisor or director	83	14.8	4.07 ± 1.04			3.67 ± 1.14		
**Employment type**				7.313	0.001		67.203	0.000
Formal	384	68.7	4.06 ± 1.09			3.32 ± 1.07		
Contracted	97	17.4	4.32 ± 0.29			4.27 ± 0.50		
Temporary	78	13.8	4.43 ± 0.33			4.35 ± 0.50		
**Hospital ownership**				21.451	0.000		0.970	0.325
Public hospital	393	70.3	4.29 ± 0.83			3.62 ± 1.02		
Private hospital	166	29.7	3.85 ± 1.08			3.65 ± 1.08		

Additionally, Table 2 details the means, standard deviations, and intervariable correlations. The results indicated a significant correlation between ethical climate types and service behavior dimensions.

**Table 2:** Descriptive statistics

Variables	Mean	S.D.	1	2	3	4	5	6	7	8	9	10	11	12	13	14	15
1. Hospital ownership	1.30	0.457	1														
2. Gender	1.06	0.246	0.085^*^	1													
3. Age (years)	2.35	0.814	− 0.166^**^	− 0.060	1												
4. Clinical tenure	1.93	1.279	− 0.103^*^	− 0.054	0.803^**^	1											
5. Education level	2.35	0.798	− 0.173^**^	0.013	0.378^**^	0.224^**^	1										
6. Professional title	1.67	0.807	− 0.202^**^	− 0.065	0.648^**^	0.630^**^	0.418^**^	1									
7. Position	1.50	0.876	0.232^**^	0.008	0.310^**^	0.219^**^	0.123^**^	0.126^**^	1								
8. Employment type	1.83	0.664	0.093^*^	− 0.085^*^	− 0.115^**^	− 0.168^**^	− 0.091^*^	− 0.247^**^	− 0.038	1							
9. Caring	3.52	1.107	0.043	− 0.018	0.373^**^	0.227^**^	0.215^**^	0.141^**^	0.339^**^	0.013	1						
10. Law and code	4.10	0.966	− 0.228^**^	− 0.074	0.478^**^	0.263^**^	0.425^**^	0.316^**^	0.088^*^	0.007	0.524^**^	1					
11. Rule	4.21	0.921	− 0.283^**^	− 0.072	0.506^**^	0.281^**^	0.451^**^	0.318^**^	0.099^*^	0.004	0.503^**^	0.856^**^	1				
12. Instrumentality	3.24	1.077	0.247^**^	0.072	0.240^**^	0.085^*^	0.124^**^	− 0.038	0.466^**^	0.052	0.496^**^	0.321^**^	0.294^**^	1			
13. Independence	3.02	1.178	0.254^**^	0.082	0.201^**^	0.070	0.159^**^	− 0.043	0.527^**^	0.052	0.517^**^	0.239^**^	0.234^**^	0.785^**^	1		
14. ISB	4.16	0.929	− 0.212^**^	− 0.027	0.507^**^	0.280^**^	0.446^**^	0.328^**^	0.150^**^	0.013	0.537^**^	0.856^**^	0.865^**^	0.339^**^	0.288^**^	1	
15. ESB	3.63	1.039	0.013	− 0.032	0.378^**^	0.195^**^	0.273^**^	0.146^**^	0.391^**^	0.008	0.579^**^	0.477^**^	0.496^**^	0.502^**^	0.527^**^	0.578^**^	1

N = 559 observations; * *p* < .05,** *p* < .01 (2-tailed)

ISB: In-role service behavior; ESB: Extra-role service behavior

### Hypothesis testing

Group comparison was used to compare the connection between ethical climate types and service behaviors of nurses in public and private hospitals. The results are shown in Table 3.

**Table 3:** Effect analysis of group comparison

Hospital ownership	Path	Indirect effects	*p*	95 % C.I.
**Public hospital**	***In-role service behavior on***			
	Caring	0.092	< 0.01	(0.027, 0.116)
	Law and code	0.287	< 0.001	(0.172, 0.421)
	Rules	0.511	< 0.001	(0.392, 0.672)
	Instrumental	0.012	0.770	(-0.058, 0.071)
	Independent	0.034	0.380	(-0.029, 0.081)
	***Extra-role service behavior on***			
	Caring	0.205	< 0.001	(0.071, 0.320)
	Law and code	0.004	0.959	(-0.196, 0.211)
	Rules	0.270	< 0.01	(0.145, 0.571)
	Instrumental	0.014	0.872	(-0.153, 0.182)
	Independent	0.235	< 0.01	(0.057, 0.344)
**Private hospital**	***In-role service behavior on***			
	Caring	− 0.047	0.564	(-0.160, 0.083)
	Law and code	0.578	< 0.001	(0.400, 0.688)
	Rules	0.365	< 0.001	(0.221, 0.493)
	Instrumental	0.019	0.855	(-0.137, 0.176)
	Independent	0.034	0.783	(-0.144, 0.193)
	***Extra-role service behavior on***			
	Caring	0.347	< 0.01	(0.106, 0.506)
	Law and code	0.091	0.461	(-0.171, 0.303)
	Rules	− 0.039	0.613	(-0.196, 0.141)
	Instrumental	0.375	< 0.01	(0.117, 0.626)
	Independent	0.029	0.851	(-0.200, 0.234)

More specifically, in public hospitals, the results showed that a caring climate significantly predicted both nurses’ in-role service behavior (*β* = 0.092; CI _95 %_ = [0.027, 0.116]; *p* < .01) and extra-role service behavior (*β* = 0.205; CI _95 %_ = [0.071, 0.320]; *p* < .001); a rules climate also significantly predicted both nurses’ in-role service behavior (*β* = 0.511; CI _95 %_ = [0.392, 0.726]; *p* < .001) and extra-role service behavior (*β* = 0.270; CI _95 %_ = [0.145, 0.571]; *p* < .01). However, a law and code climate had significant positive effects only on nurses’ in-role service behaviors (*β* = 0.287; CI _95 %_ = [0.172, 0.421]; *p* < .001), and an independent climate had significant and positive effects only on nurses’ extra-role service behavior (*β* = 0.235; CI _95 %_ = [0.057, 0.344]; *p* < .01).

Furthermore, in private hospitals, the results showed that a law and code climate significantly and positively influenced nurses’ in-role service behavior (*β* = 0.578; CI _95 %_ = [0.400, 0.688]; *p* < .001), and a rules climate also significantly predicted nurses’ in-role service behavior (*β* = 0.365; CI _95 %_ = [0.221, 0.493]; *p* < .001). Additionally, a caring climate had significant and positive effects on nurses’ extra-role service behavior (*β* = 0.347; CI _95 %_ = [0.106, 0.506]; *p* < .01), and an instrumental climate had significant and positive effects on nurses’ extra-role service behavior (*β* = 0.375; CI _95 %_ = [0.117, 0.626]; *p* < .01).

Subsequently, we tested the moderating effects of hospital ownership on ethical climate types and nurses’ service behaviors [40]. As shown in Table 4, we defined Diff = public hospital - private hospital, none of the 95 % credibility intervals included zero, suggesting that the main influence caused by a law and code climate on nurses’ in-role service behavior was significant and negative (*β* = − 0.277; CI _95 %_ = [-0.452, − 0.075]; *p* < .01), which means that in private hospitals, a law and code climate had a much greater influence on nurses’ in-role service behavior than it did in public hospitals. Furthermore, the main effect of a rules climate on nurses’ extra-role service behavior was significant and positive (*β* = 0.397; CI _95 %_ = [0.120, 0.651]; *p* < .01), which reveals that in public hospitals, the rules climate had a greater effect on nurses’ extra-role service behavior than it did in private hospitals. However, the main effect of an instrumental climate on nurses’ extra-role service behavior was significant and negative (*β* = − 0.352; CI _95 %_ = [-0.651, − 0.056]; *p* < .05), which indicates that in private hospitals, the instrumental climate had a greater effect on nurses’ in-role service behavior than it did in public hospitals.

**Table 4:** Moderating effect analysis

Diff	Path	Effects	*P*	95 % C.I.
***In-role service behavior on***				
*1*	Caring	0.114	0.079	(-0.023, 0.236)
*2*	Law and code	− 0.277	< 0.01	(-0.452, − 0.075)
*3*	Rules	0.195	< 0.05	(-0.021, 0.365)
*4*	Instrumental	− 0.009	0.921	(-0.178, 0.157)
*5*	Independent	− 0.008	0.929	(-0.179, 0.184)
***Extra-role service behavior on***				
*6*	Caring	− 0.121	0.314	(-0.348, 0.116)
*7*	Law and code	− 0.083	0.598	(-0.395, 0.210)
*8*	Rules	0.397	< 0.01	(0.120, 0.651)
*9*	Instrumental	− 0.352	< 0.05	(-0.651, − 0.056)
*10*	Independent	0.182	0.175	(-0.072, 0.441)

Diff = public hospital - private hospital

## Discussion

### Interpreting the findings

 This study is the first to compare the connection between ethical climate types and in-role and extra-role service behaviors of nurses in public and private hospitals. It confirmed that perceptions of ethical climate type influenced the extent to which nurses engaged in in-role and extra-role service behaviors. These comparisons indicated that nurses’ service behavior is a complex notion that is influenced by many organizational factors, thus contributing to the nursing service management literature.

Firstly, the outcome of the current study confirms that the caring climate and rules climate in public hospitals significantly predicted both nurses’ in-role service behavior and extra-role service behavior. Because the caring climate is linked with the construct of benevolence and the individual and local locus of analysis, nurses working in a caring climate believe that their service behaviors should be based on an overall concern for the well-being of others not only in-role behaviors but also extra-role behaviors. The results in this study are consistent with previous studies suggesting that a caring climate was significantly correlated with nurses’ ethical behaviors [27, 41]. Additionally, this conclusion is showing consistency with the literature, which indicates that a caring climate is related to positive extra-role service behavior [42]. As the rules climate is associated with the principle construct and the local locus of analysis, in this climate, it is generally accepted that nurses’ service decisions are guided by a strong and universal set of local rules or standards, such as codes of conduct [43–45]. Moreover, the rules climate was found to have a greater effect on nurses’ service behaviors than a caring climate. This result is in line with Abou’s [36] study, which showed that nurses believed that the most frequent or common ethical climate is rules guided. A possible explanation might be that most nurses perceived that the rules climate was significant and that “rules and procedures are to be strictly followed” [46].

Second, the law and code climate only significantly influenced nurses’ in-role service behavior in both public and private hospitals. A law and code climate involves an organization that supports principled decision-making based on external criteria, which includes laws or professional codes of conduct. Hence, we found that nurses in a law and code climate tend to devote themselves to their professional duties and provide more in-role service behavior.

Moreover, it was found that nurses in public hospitals rather than private hospitals in the independent climate acted more extra-role service behaviors. Because in an independent climate, nurses acted according to the personal moral beliefs of their own based on their set of well-thought-out principles and served patients in a way that exceeded their formal job requirements. Therefore, based on the above findings, we suggest that managers in public hospitals should focus on the development of caring, rules, law and code, and independent climates. It is particularly important to address the caring and rules climates.

 Third, it was found that private hospital nurses provided more in-role service behavior if they perceived a greater law and code and rules climate, which is consistent with the results in public hospitals outlined above. We can conclude from these results that the existence of an ethical climate type of law and code in both public and private hospitals is in line with the idea of promoting the public interest, as this type emphasizes the principles of fairness, accountability, transparency, and equality [47, 48]. All nurses should be provided with hospital education and training courses so that they are aware of and have knowledge of the guidelines and rules formulated by professional institutions or laws promulgated by the government. Nurses will thus provide more in-role service behavior and satisfy patients.

Meanwhile, it was found that private hospital nurses provided more extra-role service behavior if they perceived more of a caring and instrumental climate. In a caring climate, the main consideration is how to maximize the interests of everyone in the organization, and nurses in both public and private hospitals who feel a caring climate will integrate themselves into their work and in turn provide additional services to their patients [49]. One important finding from the research suggests that although in an instrumental environment, the goal of the first importance is to provide the interests of the organization or to serve personal benefit with little regard for the needs and interests of others [36], private hospital nurses perceiving a more instrumental climate are willing to exhibit greater extra-role service behavior, which is in contrast with Leung’s [42] finding that an instrumental climate is associated with negative extra-role behaviors. A possible explanation may be that private hospital nurses provide additional services to patients spontaneously, which implies more extra-role service behaviors that benefit patients and thus contribute to nurses’ performance. This observation is consistent with Simha and Cullen’s [50] idea that the instrumental climate encourages employees to seek out better prospects for themselves and has a significant positive influence on nurses’ continuance commitment [51].

Lastly, when comparing the influences of ethical climate types on nurses’ service behavior in different ownership hospitals, our research indicates that in private hospitals, the law and code climate had a much greater effect on nurses’ in-role service behavior than it did in public hospitals. It is recommended that hospital authorities in China establish comprehensive legal and professional rules and internalize them more effectively in private hospitals so that the law and code climate can have a greater impact on the in-role service behavior of nurses in private hospitals. In addition, findings from the study indicate that the instrumental climate had a greater effect on private hospital nurses’ extra-role service behavior than it did in public hospitals. According to Victor and Cullen [19], the instrumental climate is closely linked to egoistic structures and personal and local points of analysis. As a result, nurses working in the instrumental climate tend to perceive their organization as encouraging norms of ethical decision-making and expectations from an egoistic perspective. Especially for private hospital nurses, an instrumental climate would encourage them to provide much greater extra-role service behavior to facilitate their job performance and seek better prospects for themselves. However, the conclusions of this study demonstrated that the rules climate had a greater effect on public hospital nurses’ extra-role service behavior than on those in private hospitals. Because of the close link between the rules climate and the accepted rules of conduct defined by the organization, we can conclude that the rules climate ensures that public hospital nurses strictly adhere to hospital rules and procedures and perform extra-role service behaviors.

### Implications for nursing management

Understanding the relations between the types of ethical climate and the dimensions of service behavior is conducive to the generation of incentive strategies for nurses. As hospital ownership has an impact on the quality of nursing service, the findings provide a piece of clear and valuable information about how public and private hospital administrators motivate nurses to deliver high-quality care and service. The caring and instrumental climate are the key to promote extra-role service behavior for nurses in private hospitals. And the independent climate has a great effect on extra-role service behaviors for nurses in public hospitals.

### Limitations

There are some limitations of this study that may affect the results. One potential limitation is that all variables are measured by self-report, which may suffer from response bias in each respondent [52]. Second, this study experienced a variable-centered method that presumes that employees provide all aspects of service behaviors equally. This research approach is similar to previous studies testing service behavior. In fact, various dimensions play a variety of roles. A person-centered approach can use latent trait surveys to gain insight into service behaviors, in other words, to understand the typology of service behaviors. Lastly, this study focused primarily on the moderating effect of hospital ownership on the connection between the service behavior dimensions and the type of ethical climate. Future research should examine the psychological process that how ethical climate affects nurses’ service behaviors and also explore the interaction effect of individual and organizational factors on service behaviors of nurses.

## Conclusions

The outcomes of this study indicate that a caring and rules climate in public hospitals significantly predicted both nurses’ in-role service behavior and extra-role service behavior. In addition, an independent climate only influenced their extra-role service behavior. In regard to private hospitals, if nurses perceived a greater law and code and rules climate, they provided more in-role service behavior, and if they perceived a greater caring and instrumental climate, they provided more extra-role service behavior. In comparing the two ownership hospitals, our research indicates that (1) in private hospitals, the law and code climate had a much greater influence on nurses’ in-role service behavior than it did in public hospitals, and (2) the instrumental climate had a greater effect on private hospital nurses’ extra-role service behavior than on those in public hospitals; however, (3) the rules climate had a greater effect on public hospital nurses’ extra-role service behavior than on those in private hospitals.

## Supplementary Information

**Additional file 1.**

## Data Availability

The datasets used and analyzed during the current study are confidential, but they will be available from the corresponding author on reasonable request.

## Abbreviations

ISB: In-role service behavior
ESB: Extra-role service behavior

## References

[1] Azimzadeh R, Valizadeh L, Zamanzadeh V, Rahmani A (2013). What are important for patient centered care? A quantitative study based on perception of patients’ with cancer. J Caring Sci.

[2] Michie S, Miles J, Weinman J (2003). Patient-centredness in chronic illness: what is it and does it matter?. Patient Educ Couns.

[3] Cole FL, Mackey TA, Lindenberg J (2010). Wait time and satisfaction with care and service at a nurse practitioner managed clinic. J Am Acad Nurse Practitioners.

[4] Knudtson N (2010). Patient satisfaction with nurse practitioner service in a rural setting. J Am Acad Nurse Pract.

[5] Podsakoff NP, Whiting SW, Podsakoff PM, Blume BD (2009). Individual- and organizational-level consequences of organizational citizenship behaviors: a meta-analysis. J Appl Psychol.

[6] Laine C, Davidoff F (1996). Patient-centered medicine: a professional evolution. JAMA.

[7] Lützén K, Dahlqvist V, Eriksson S, Norberg A. Developing the concept of moral sensitivity in health care practice. Nurs Ethics. 2006;13(2):187–96. 10.1191/0969733006ne837oa 16526152

[8] Zhang N, Li M, Gong Z, Xu D. Effects of ethical leadership on nurses’ service behaviors. Nurs Ethics. 2019;26(6):1861–72. 10.1177/0969733018787220 30078367

[9] Kang D, Stewart J, Kim H, Lim J. Unravelling the impact of psychological empowerment on customer service behaviours as a consequence of ‘leader-member exchange.’ Serv Ind J. 2011;32(11):1791–809.

[10] Lee YK, Nam JH, Park DH, Lee KA. What factors influence customer-oriented prosocial behavior of customer‐contact&nbsp;employees? J Serv Mark. 2006;20(4):251–64.

[11] Tsaur S, Wang C, Yen C, Liu Y (2014). Job standardization and service quality: the mediating role of prosocial service behaviors. Int J Hosp Manag.

[12] Raub S, Robert C (2010). Differential effects of empowering leadership on in-role and extra-role employee behaviors: exploring the role of psychological empowerment and power values. Hum Relat.

[13] Cheng J, Chen C (2017). Job resourcefulness, work engagement and prosocial service behaviors in the hospitality industry. Int J Contemp Hosp M.

[14] Bettencourt LA, Gwinner KP, Meuter ML (2001). A comparison of attitude, personality, and knowledge predictors of service-oriented organizational citizenship behaviors. J Appl Psychol.

[15] Groth M (2005). Customers as good soldiers: examining citizenship behaviors in internet service deliveries. J Manage.

[16] Organ DW (1997). Organizational citizenship behavior: it’s construct clean-up time. Hum Perform.

[17] Kim SKI, Zhan Y, Hu X, Yao X. Effects of customer entitlement on employee emotion regulation, conceding service behaviour, and burnout: the moderating role of customer sovereignty belief. Eur J Work Organ Psy. 2020(4):1–17.

[18] Victor B, Cullen JB (1987). A theory and measure of ethical climate in organizations. Res Corporate Soc Performance Policy.

[19] Victor B, Cullen JB (1988). The organizational bases of ethical work climates. Admin Sci Quart.

[20] Rodney P, Doane GH, Storch J, Varcoe C (2006). Toward a safer moral climate. Canadian Nurse.

[21] Lützén K, Blom T, Ewalds-Kvist B, Winch S. Moral stress, moral climate and moral sensitivity among psychiatric professionals. Nurs Ethics. 2010;17(2):213–24. 10.1177/0969733009351951 20185445

[22] Numminen O, Leino-Kilpi H, Isoaho H, Meretoja R (2015). Ethical climate and nurse competence-newly graduated nurses’ perceptions. Nurs Ethics.

[23] Hall LM, Doran D (2004). Nurse staffing, care delivery model, and patient care quality. J Nurs Care Qual.

[24] Kia N, Halvorsen B, Bartram T. Ethical leadership and employee in-role performance: the mediating roles of organisational identification, customer orientation, service climate, and ethical climate. Pers Rev. 2019 2019-01-01;48(7):1716–33.

[25] Tehranineshat B, Torabizadeh C, Bijani M. A study of the relationship between professional values and ethical climate and nurses’ professional quality of life in iran. International Journal of Nursing Sciences. 2020 2020-01-01;7(3):313–9. 10.1016/j.ijnss.2020.06.001 PMC7424154 32817854

[26] Bell SE (2003). Ethical climate in managed care organizations. Nurs Adm Q.

[27] Fu W, Deshpande SP. The impact of caring climate, job satisfaction, and organizational commitment on job performance of employees in a china’s insurance company. J Bus Ethics. 2013;2013-09–03:1–11.

[28] Pan J, Zhao H, Wang X, Shi X (2016). Assessing spatial access to public and private hospitals in sichuan, china: the influence of the private sector on the healthcare geography in china. Soc Sci Med.

[29] Cooke FL, Zhan C. Between market and bureaucracy: public healthcare reforms in china and nurses’ terms and conditions. Int J Hum Resour Man. 2013 2013-01-01;24(16):3178–95.

[30] Ye L, Zhang X, Lai X (2017). Does hospital ownership influence hand hygiene compliance?. Curr Med Sci.

[31] Chen L, Dai Y, Zhang Y, Wu Q, Rudan D, Saftić V (2013). A comparison between antenatal care quality in public and private sector in rural hebei, china. Croat Med J.

[32] Eggleston K, Lu M, Li C, Jian W, Zhe Y, Jing Z (2010). Comparing public and private hospitals in china: evidence from guangdong. Bmc Health Serv Res.

[33] Maun A, Wessman C, Sundvall PD, Thorn J, Björkelund C (2015). Is the quality of primary healthcare services influenced by the healthcare centre’s type of ownership? —an observational study of patient perceived quality, prescription rates and follow-up routines in privately and publicly owned primary care centres. Bmc Health Serv Res.

[34] Zhang X, Wen D, Liang J, Lei J (2017). How the public uses social media wechat to obtain health information in china: a survey study. Bmc Med Inform Decis Making.

[35] Brislin RW (1970). Back-translation for cross-cultural research. J Cross Cult Psychol.

[36] Abou Hashish EA (2017). Relationship between ethical work climate and nurses’ perception of organizational support, commitment, job satisfaction and turnover intent. Nurs Ethics.

[37] Cullen JB, Victor B, Bronson JW. The ethical climate questionnaire: an assessment of its development and validity. Psychol Rep. 1993 1993-10-01;73(2):667–74.

[38] Chen H. A study of the relationships between orientation training, service behavior, and job performance of the newly hired nurses in veteran hospital [Mater dissertation]. Taiwan: National Chi Nan University; 2010.

[39] Zhang N, Gong Z, Xu Z, Gilal FG (2019). Ethical climate and service behaviours in nurses: the moderating role of employment type. J Adv Nurs.

[40] Hess JD, Ye H, Blair E. On testing moderation effects in experiments using logistic regression. SSRN Electronic Journal. 2014 2014-02-10. Available at SSRN: https://ssrn.com/abstract=2393725.

[41] Deshpande SP, Joseph J (2009). Impact of emotional intelligence, ethical climate, and behavior of peers on ethical behavior of nurses. J Bus Ethics.

[42] Leung ASM (2008). Matching ethical work climate to in-role and extra-role behaviors in a collectivist work setting. J Bus Ethics.

[43] Aquino K, Becker TE (2010). Lying in negotiations: how individual and situation factors influence the use of neutralization strategies. J Organ Behav.

[44] Liu AMM, Fellows R, Ng J (2004). Surveyors’ perspectives on ethics in organisational culture. Eng Construct Architect Manage.

[45] Martin KD, Cullen JB (2006). Continuities and extensions of ethical climate theory: a meta-analytic review. J Bus Ethics.

[46] Filipova AA (2011). Ethical climates in for-profit, nonprofit, and government skilled nursing facilities. JONA’ S Healthcare Law. Ethics Regul.

[47] Malloy DC, Agarwal J (2010). Ethical climate in government and nonprofit sectors: public policy implications for service delivery. J Bus Ethics.

[48] Rosario Laratta (2009). Ethical climate in nonprofit organizations: a comparative study. Int J Sociol Soc Policy.

[49] Karatepe O (2013). Inking perceived ethical climate to performance outcomes: the mediating role of job embeddedness. Ekonomska Istraživanja-Economic Research.

[50] Simha A, Cullen JB (2012). Ethical climates and their effects on organizational outcomes: implications from the past and prophecies for the future. Acad Manage Perspect.

[51] Tsai M, Huang C. The relationship among ethical climate types, facets of job satisfaction, and the three components of organizational commitment: a study of nurses in taiwan. J Bus Ethics. 2008 2008-01-01;80(3):565–81.

[52] Polit DF, Beck CT. Nursing research: principles and methods. 7th edition ed. Philadelphia, PA: Lippincott Williams & Wilkins; 2004.

