# Supplementary material for: The relationship between ethical climate and nursing service behavior in public and private hospitals: a cross-sectional study in China
Source: BMC Nurs. 2021 Aug 5;20:136. doi: 10.1186/s12912-021-00655-7 (PMC8339387; doi:10.1186/s12912-021-00655-7)
Supplement: Supplementary file 1 — Additional file 1. [file 12912_2021_655_MOESM1_ESM.doc]

**Demographic Characteristics**

1. Gender □Female □Male

2. Clinical tenure (years) □≤ 5 □6-10 □11-15 □16-20 □> 20

3. Age □≤ 20 □21-30 □31-40 □> 40

4. Education level □Certificate (technical school) □Junior College

□Bachelor’s degree □Master’s degree or above

5. Professional title □Primary nurse □Junior nurse □Senior nurse

6. Position □General nurse □Unit manager □Supervisor or director

7. Employment type □Formal stuff □Contracted stuff □Temporary stuff

8. Hospital ownership □Public hospital □Private hospital

**Ethical Climate Questionnaire**

**Caring**

1. Our major consideration is what is best for everyone in the hospital.

2. The most important concern is the good of all the people in the hospital.

3. In this hospital, our major concern is always what is best for the other person.

4. In this hospital, people look out for each other’s good.

5. It is expected that you will always do what is right for the patients and public.

**Law and professional**

6. People are expected to comply with the law and professional standards over and above other considerations.

7. In this hospital, the law or ethical code of their profession is the major consideration.

8. In this hospital, people are expected to strictly follow legal or professional standards.

9. The first consideration is whether a decision violates any law.

**Rules**

10. It is very important to follow strictly the hospital’s rules and procedures here.

11. Everyone is expected to stick by hospital rules and procedures.

12. Successful people in this hospital go by the book.

13. Successful people in this hospital strictly obey the hospital policies.

**Instrumental**

14. In this hospital, people protect their own interest above other considerations.

15. In this hospital, each person is expected, above all, to work efficiently.

16. There is no room for one’s own personal morals or ethics in this hospital.

17. The most efficient way is always the right way, in this hospital.

18. People are concerned with the hospital’s interests-to the exclusion of all else.

19. Work is considered sub-standard only when it hurts the hospital’s interests.

**Independence:**

20. People are expected to do anything to further the hospital’s interests.

21. In this hospital, people are expected to follow their own personal and moral beliefs.

22. Each people in this hospital decides for himself what is right and wrong.

23. The most important consideration in this hospital is each person’s sense of right and wrong.

24. In this hospital, people are guided by their own personal ethics.

**Nurses’** **Service Behaviour Scale**

**Role-prescribed service behaviour:**

1. I can provide nursing services according to the job responsibilities prescribed by the hospital.
2. I am familiar with the service procedures of different work shifts.
3. I can independently fulfill responsibilities to patients as specified in the nursing job description.
4. I can satisfy the needs of patients with pleasure in my duties.
5. I have good manners as a nurse with a neat and professional appearance.

**Extra-role service behaviour:**

1. I can help patients to solve problems beyond what is expected or required of the nursing work contents.
2. When the patient is in need, I can provide additional services voluntarily.
3. Even beyond my job requirements, I take the initiative to meet the needs of the patients.
